# Trend and disparities in authorship of healthcare-related publications on the ongoing Russia-Ukraine war

**DOI:** 10.1186/s12939-023-02070-7

**Published:** 2023-12-12

**Authors:** Habib Olatunji Alagbo, Saloni Mitra, Karen Madueke, Uchechi Blessing Azuwike, Samantha Dos Santos Rocha Ferreira, Alimat Temitope Ademuyiwa, Oluwaseun Adeleke, Chigozirim Ejinkeonye, David Izuchukwu Onyebuchi, Inioluwa Atowoju, Faith Inioluwa Odelola, Jyoti Kumari, Marvellous Sowunmi, Yana Al-Inaya, Toufik Abdul-Rahman, Nathan A. Shlobin

**Affiliations:** 1 School of Medicine, V.N. Karazin National University, Kharkiv, Ukraine; 2 International Students Surgical Network (InciSioN) Ukraine, Kiev, Ukraine; 3 OO Bogomolets National Medical University, Kiev, Ukraine; 4 Faculty of Medicine, University of Novi Sad, Novi Sad, Serbia; 5 Immanuel Kant Baltic Federal University, Kaliningrad, Russia; 6 Medical Institute, Sumy state university, Sumy, Ukraine; 7 Petre Shotadze Tbilisi Medical Academy, Tbilisi, Georgia; 8 Kharkiv National Medical University, Kharkiv, Ukraine; 9 Medical Faculty, National Research Ogarev Mordovia State University, Saransk, Russia; 10 Uzhhorod National University, Uzhhorod, Ukraine; 11 Northwestern University Feinberg School of Medicine, Chicago, IL USA

**Keywords:** Armed Conflict, Authorship, Ukraine, Russia

## Abstract

**Background:**

The Russia-Ukraine war has undeniably impacted global science and healthcare in Ukraine. Many Ukrainian researchers have had their projects disrupted by this war, either due to loss of life, displacement, or destruction of resources. Despite these challenges, these researchers have sought to make their voices heard. This scoping review highlights the trend of healthcare-related publications on the current Russia-Ukraine war and characterizes the contribution of Ukrainian authors to these publications.

**Method:**

A comprehensive literature search was performed using two databases (Scopus and Pubmed) for publications related to the ongoing Russia-Ukraine war. We included articles only related to healthcare. We then extracted and analyzed bibliometric data.

**Result:**

One hundred and eighty-three articles were identified, including 12 (6.6%) original articles, 26 (14.2%) cross-sectional studies, 19 (10.4%) letters to the editor, 10 (5.5%) commentaries, 5 (2.7%) perspectives, 35 (19.1%) editorials, 2 (1.1%) randomized controlled trials, 11(6.0%) correspondences, 13 (7.1%) opinions, 8 (4.4%) reviews and 42 (23.0%) are identified as others. 180 (98.4%) studies were in English, and 3 (1.7%) were in German. 54 (29.5%) papers on the war had at least one author affiliated with a Ukrainian institution, and 29 (15.9%) studies had authors with Ukrainian affiliation as first authors.

**Conclusion:**

our study shows that there has been a significant number of publications on the Russia-Ukraine war and only a small portion of first authors, co-authors, and last authors of these publications are affiliated to an institution in Ukraine. Therefore, despite the relatively high number of publications, most publications do not arise from the perspective of Ukrainian authors.

**Supplementary Information:**

The online version contains supplementary material available at 10.1186/s12939-023-02070-7.

## Introduction

It has been over a year since the Russia-Ukraine war began, and this war has led to high civilian casualties in the country, resulting in 9,369 deaths and 16,646 injured individuals [1]. The Russia-Ukraine war has brought forth numerous challenges, including a significant loss of human life, displacement, destruction of infrastructures, extensive damage to productive capacity (including electricity loss), and a reduction of private consumption by over a third compared to pre-war levels [2].

Apart from the various setbacks the country has faced, the war has also significantly impacted Ukraine’s scientific contributions in healthcare [3, 4]. Many health workers in Ukraine ceased patient care following the war outbreak, and others were compelled to abandon clinical facilities due to targeted attacks on the health system [5, 6]. As a result, a considerable portion of the health workforce either left the country or became internally displaced, resulting in a substantial reduction at a time when their services are most critically needed [5]. Furthermore, the war’s repercussions have compelled both national and foreign medical students to forsake their studies and pursue training abroad, leading to a notable decrease in the number of health workers graduating and practicing within Ukraine’s healthcare system [5]. Despite facing challenges such as migration, adapting to a new environment, and the mental health toll [7], Ukrainian authors have persisted in disseminating information about the ongoing Russia-Ukraine war to ensure their perspectives are heard. Numerous Ukrainian healthcare researchers and authors have published articles highlighting the repercussions and scope of the ongoing war on health in their country, illuminating how the Russian attack has detrimentally affected Ukraine [8].

Underrepresentation of authors from Low- and middle-income countries (LMICs) as first and last authors of publications about LMICs have been reported in the literature [9]. This underrepresentation can further be aggravated in situations of conflict, a time at which it is necessary that the narrative of the impact of the situation on healthcare be driven by local researchers due to their first-hand experience and their profound knowledge of the situation on ground. But, despite the resilience of Ukrainian authors and their continuous efforts to ensure the impact of the war on healthcare is documented in the literature, it is probable that their contribution to the literature on the ongoing war might not be significant enough, hence, the purpose of our study, to assess the contribution of Ukrainian authors to the publications on the Russia-Ukraine war.

## Methods

### Literature search

A comprehensive literature search of two databases (PubMed and Scopus) was performed for articles published on the current Russia-Ukraine war with a focus on the ones related to healthcare, from the start of the war in February 2022 up till February 2023 inclusively. Major keywords such as “war”, “conflict”, “invasion”, “Ukraine”, “Russia” etc. (see appendix 1 for search strategy) were used to identify articles relevant to our research. The Preferred Reporting Items for Systematic Reviews and Meta-Analyses (PRISMA) guidelines for scoping reviews (PRISMAScR) were applied [10].

All articles were screened by two independent reviewers at the abstract and full-text screening stages and any conflict was resolved by a third independent reviewer. The inclusion criteria were (1) articles published from February 2022 inclusive, (2) articles on the ongoing Russia-Ukraine war, (3) articles related to healthcare, (4) articles which full-text is available, and (5) no restriction on language. The exclusion criteria were (1) articles published before February 2022, (2) not related to the ongoing Russia-Ukraine war, (3) not related to healthcare, (4) with no authors’ affiliation, (5) not peer-reviewed, and (6) with full text is not available.

### Data synthesis and analysis

Bibliometric data such as date of publication (month and year), journal, article type, affiliation of the authors (whether any author and the proportion of authors affiliated with Ukraine), language of publication, and article specialty (whether they are in public health, psychiatry, or specific surgical or medical subspecialty such as nephrology, orthopaedic surgery, etc.) were extracted and analyzed using Excel (Microsoft Inc., Redmond, Washington, USA) to identify the trend in publication and authorship distribution in the first 12 months of the war [11]. The first and last authorship was specifically analyzed because of the traditional importance placed on this position [12]. The type and specialty of the articles were determined based on what was indicated in the publication or was determined by a team led by the senior researcher (HOA) if unavailable in the publication. Articles whose specific type or specialty could not be determined were categorized as others.

We considered authors unique for each included manuscript and analyzed, and for articles with a single author, the author was considered both as first and last author for that manuscript in our analysis.

## Results

**Fig. 1:**
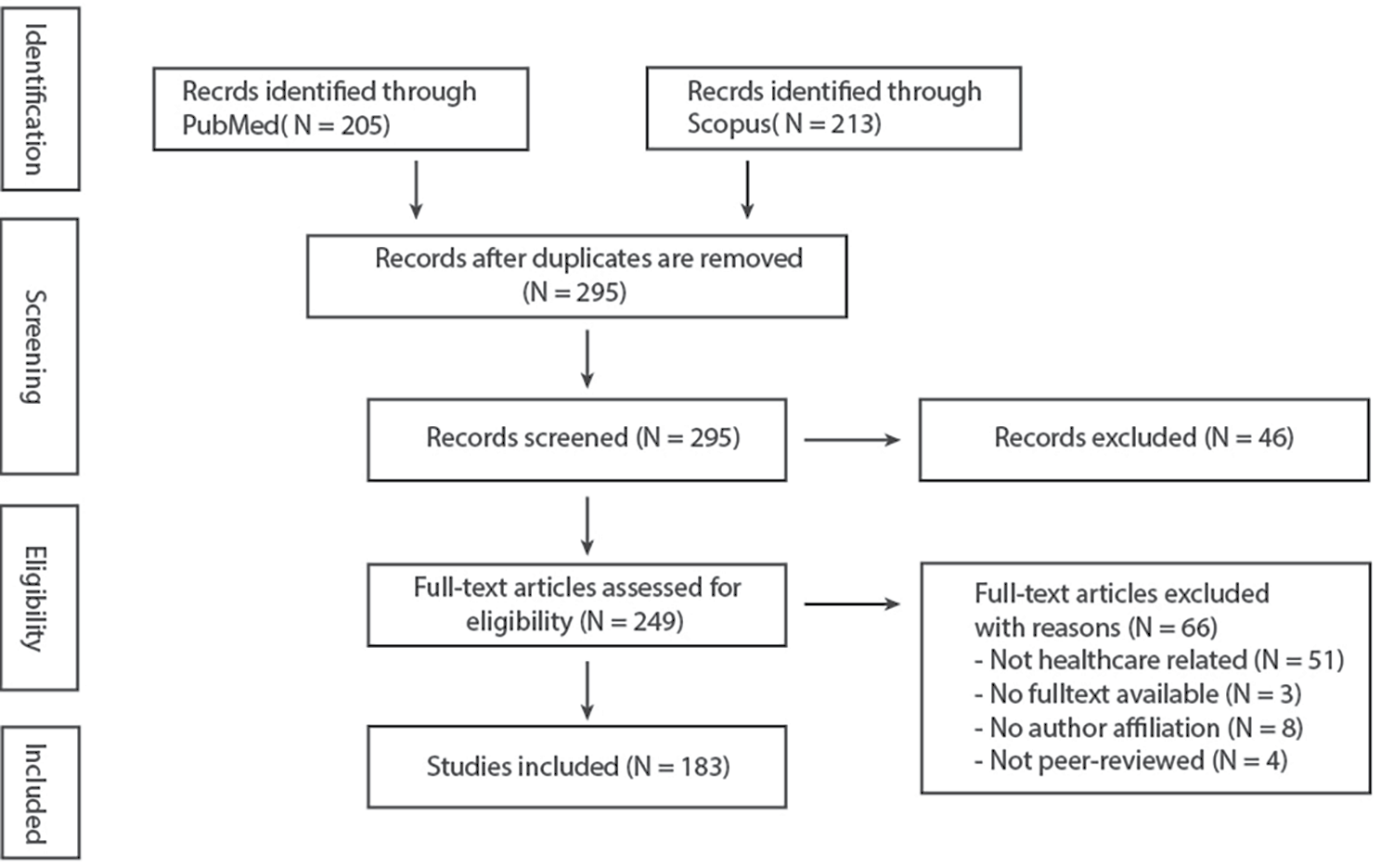
PRISMA Flow Diagram

**Fig. 2:**
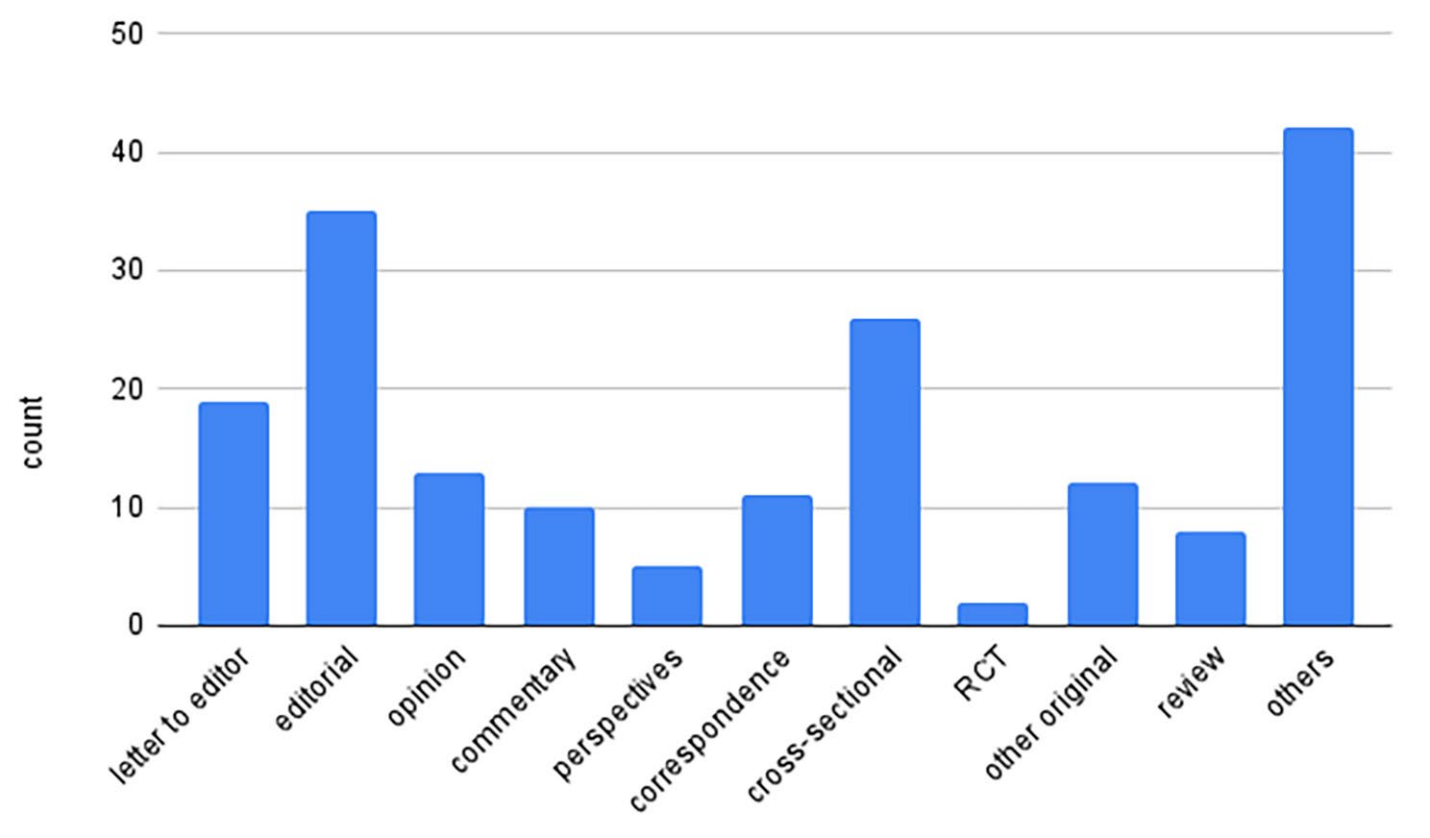
Types of Articles

**Fig. 3:**
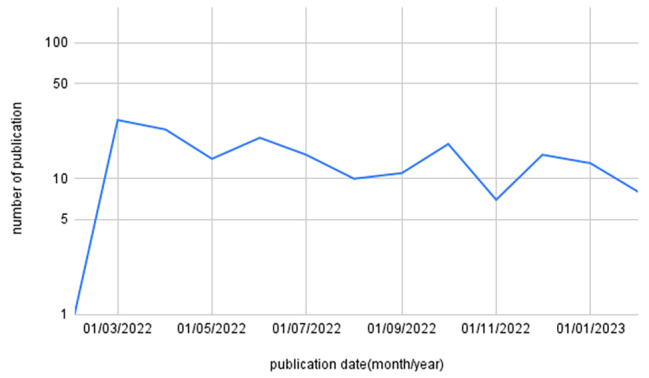
Publications over time

A total of 419 publications were identified in the literature search, with a total of 122 duplicates removed (Fig. 1: PRISMA Flow Diagram). After screening, 183 articles were included in the final analysis. The types of articles included 19 (10.4%) letter to the editor, 35 (19.1%) editorial, 13 (7.1%) opinions, 10 (5.5%) commentaries, 5 (2.7%) perspectives, 11 (6.0%) correspondence, 26 (14.2%) cross-sectional studies, 2 (1.1%) randomized controlled trials (RCTs), 12 (6.6%) other types of original research, 8 (4.4%) reviews, and 42 (23.0%) articles categorized as others (Fig. 2: Types of Articles). The language distribution of these publications revealed that the majority, 180 (98.4%) were in English, and 3 (1.6%) were in German. In the year since the war began, most articles were published in March 2022 (n = 27, 14.8%), April (n = 23, 12.6%), June (n = 20, 11.0%), and October (n = 18, 9.9%) (Fig. 3: Publications over time).

International Journal of Environmental Research and Public Health (n = 9, 4.9%), The Lancet (n = 9, 4.9%), BMJ (Clinical Research ed.) (n = 7, 3.8%), and Asian Journal of Psychiatry (n = 5, 2.7%) were the journals with the most publications. The specialty of the articles published was mostly public health (n = 53, 32.2%), followed by Psychiatry (n = 20, 10.9%), and nephrology (n = 5, 2.7%), with specialties categorized as others coming in 4th (n = 10, 5.5%).

**Fig. 4:**
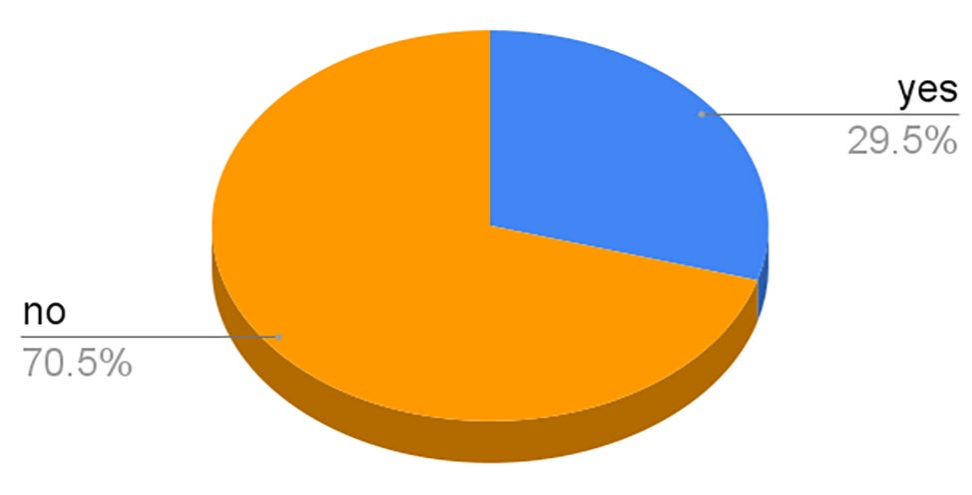
At least one author is Ukraine affiliated

Regarding authorship, 54 (29.5%) of the publications had at least one Ukrainian author, while 129 (70.5%) did not have any author affiliated with Ukraine (Fig. 4: At least one author is Ukraine affiliated). In terms of articles with their first or last author affiliated with Ukraine, 29 (15.9%) had their first author affiliated with Ukraine, while 28 (15.3%) had their last author affiliated with Ukraine (Table 1: Articles with first or last author affiliated with Ukraine). Also, 138 (75.4%) articles have 0–25% of authors affiliated to Ukraine, 18 (9.8%) have 26–50% affiliated to Ukraine, 7 (3.8%) have 51–75% of authors affiliated to Ukraine while 20 (10.9%) have 76–100% of its authors affiliated to Ukraine.

**Table 1:** Articles with First Author or Last Author affiliated with Ukraine

	First Author Affiliated with Ukraine N = 183	Senior Author Affiliated with Ukraine N = 183
Yes	29 (15.9%)	28 (15.3%)
No	154 (84.1%)	155 (84.7%)

## Discussion

### Summary

Our study indicates that while there is a significant amount of publication within one year of the beginning of the Russia-Ukraine war, Ukrainian authors are underrepresented both as first authors, last authors, and co-authors. These findings are particularly concerning given that the war directly impacts Ukraine, and the voices of its researchers are crucial for a comprehensive understanding of the war’s effects on healthcare.

Our findings further support findings from previous studies published on output of local researchers during conflicts or political instabilities. For example, Rema A. et al. showed that 59.3% of articles published during the period of conflict in Syria had no author affiliated to a Syrian institution [13], which is also similar to the findings of a bibliometric analysis conducted in ivory coast showing a low representation of Ivorian authors as both first and last authors during period of political instability [14].

### Underrepresentation of Ukrainian authors

The underrepresentation of Ukrainian authors in the scientific literature on the Russia-Ukraine war is a multifaceted issue. One of the primary challenges is the death and migration of researchers and medical personnel due to the ongoing conflict, which has resulted in a shortage of healthcare manpower and disrupted research activities [3]. It is important to note that research is not the focus of healthcare personnel in Ukraine given more imminent clinical needs. The lack of adequate resources for scientific translation into English also poses a significant barrier, as most of the publications are in English.

The underrepresentation of Ukrainian authors not only limits the diversity of perspectives but also risks skewing the narrative and understanding of the war’s impact on healthcare. Local researchers can provide unique insights into the challenges and needs of the healthcare system, which are essential for effective interventions and policymaking [15]. Therefore, it is crucial to include more Ukrainian voices in the global scientific discourse on the Russia-Ukraine war.

### Proposed action steps

To address this underrepresentation, several steps can be taken. First, international organizations and journals could offer translation services to Ukrainian researchers to help them publish their work. Second, special issues or sections could be dedicated to research conducted by Ukrainian authors on the impact of the war on healthcare. Third, collaboration between Ukrainian researchers and international teams could be encouraged to ensure a more balanced representation [3]. Lastly, funding agencies could prioritize grants for research focusing on the Russia-Ukraine war that includes Ukrainian researchers [6]. Additionally, we recommend that future research should focus on the quality and impact of publications by Ukrainian authors to provide a more comprehensive picture of their contribution. Studies could also explore the barriers faced by Ukrainian researchers in more detail, through surveys or interviews, to identify specific challenges and potential solutions.

### Study limitations

Our study has some limitations to be considered despite our efforts to conduct a comprehensive scoping review. Firstly, the literature search was restricted to only two databases, therefore the possibility of excluding relevant studies from other databases, as well as non-English studies, exists. The findings of this study may not be generalizable to other fields as only articles related to healthcare were included. Lastly, the study does not explore the quality or impact of the publications, which could provide further insights into the contribution of Ukrainian authors.

## Conclusion

Since February 2022, the Russia-Ukraine War has influenced the lives of millions both within and outside Ukraine. As the events of the war and its profound effects continue to unfold, it is essential that these effects, especially on healthcare, are documented by those most directly involved; that is to say, by those affiliated with Ukraine. However, there is underrepresentation of Ukrainian authors and affiliates either as first authors, last authors, or co-authors. We advocate that greater effort be put into ensuring that Ukrainian voices are heard.

### Electronic supplementary material

Below is the link to the electronic supplementary material.

Supplementary Material 1

## Data Availability

No new data was generated, but the specific data from this article will be shared upon reasonable request from the corresponding author.
