# Supplementary material for: Trend and disparities in authorship of healthcare-related publications on the ongoing Russia-Ukraine war
Source: Int J Equity Health. 2023 Dec 12;22:258. doi: 10.1186/s12939-023-02070-7 (PMC10714528; doi:10.1186/s12939-023-02070-7)
Supplement: Supplementary file 1 — Supplementary Material 1 [file 12939_2023_2070_MOESM1_ESM.docx]

Appendix 1: Search Strategies

| Pubmed Search Strategy (filters used - last one year) | | |
| --- | --- | --- |
| 1 | war | 60,439 |
| 2 | conflict | 2,682,877 |
| 3 | invasion | 767,684 |
| 4 | dispute | 20,489 |
| 5 | 1 or 2 or 3 or 4 | 3,409,768 |
| 6 | ukraine | 40,662 |
| 7 | ukrainian | 24,101 |
| 8 | 6 or 7 | 57,683 |
| 9 | russia | 204,685 |
| 10 | russian | 795,870 |
| 11 | 9 or 10 | 865,457 |
| 12 | 5 and 8 and 11 | 205 |

| Scopus Search Strategy (filters used - year [2022 - 2023], subject area [medicine]) | | |
| --- | --- | --- |
| 1 | war | 364,430 |
| 2 | conflict | 462,572 |
| 3 | invasion | 412,056 |
| 4 | dispute | 75,031 |
| 5 | 1 or 2 or 3 or 4 | 1,243,590 |
| 6 | ukraine | 63,035 |
| 7 | ukrainian | 23,517 |
| 8 | 6 or 7 | 74,311 |
| 9 | russia | 215,253 |
| 10 | russian | 326,176 |
| 11 | 9 or 10 | 426,552 |
| 12 | 5 and 8 and 11 | 213 |
